# Self-avoiding walk, spin systems and renormalization

**DOI:** 10.1098/rspa.2018.0549

**Published:** 2019-01-23

**Authors:** Gordon Slade

**Affiliations:** Department of Mathematics, University of British Columbia, Vancouver, BC, Canada V6T 1Z2

**Keywords:** self-avoiding walk, *φ*^4^ model, renormalization, critical exponent

## Abstract

The self-avoiding walk, and lattice spin systems such as the *φ*^4^ model, are models of interest both in mathematics and in physics. Many of their important mathematical problems remain unsolved, particularly those involving critical exponents. We survey some of these problems, and report on recent advances in their mathematical understanding via a rigorous non-perturbative renormalization group method.

## 1. Introduction

The self-avoiding walk (SAW) is a combinatorial model of lattice paths without self-intersections. In addition to its intrinsic mathematical interest, it arises in polymer science as a model of linear polymers, and in statistical mechanics as a model that exhibits critical behaviour. The mathematical problems associated with the SAW are notoriously difficult and there remain longstanding unsolved problems that are central to the subject. A closely related model is the weakly self-avoiding walk (WSAW), which is predicted to exhibit the same critical behaviour as the SAW.

The critical behaviour of the SAW or WSAW is expressed in terms of critical exponents, which have a qualitative and quantitative relationship with the critical exponents in models of ferromagnetism including the Ising and |*φ*|^4^ spin models. Within physics, the critical exponents are well understood, but they nevertheless present deep mathematical problems.

This article is a review of recent mathematical results about critical exponents for the WSAW and |*φ*|^4^ models, with focus on the critical behaviour of the susceptibility. Some background on the SAW and Ising models is provided for motivation and context. The results we present involve a unified treatment of the WSAW and |*φ*|^4^ models, via an exact relation between the WSAW and a ‘zero-component’ |*φ*|^4^ model. The proofs are based on a rigorous version of Wilson's renormalization group (RG) approach. We provide an introduction to the RG method from the perspective of a mathematician.

## 2. Self-avoiding walk

We discuss the SAW and WSAW models, as well as their long-range versions. The emphasis is on the critical behaviour, particularly for the susceptibility.

### (a) Strictly self-avoiding walk

#### (i) Universality and scale invariance.

**Figure 1.**
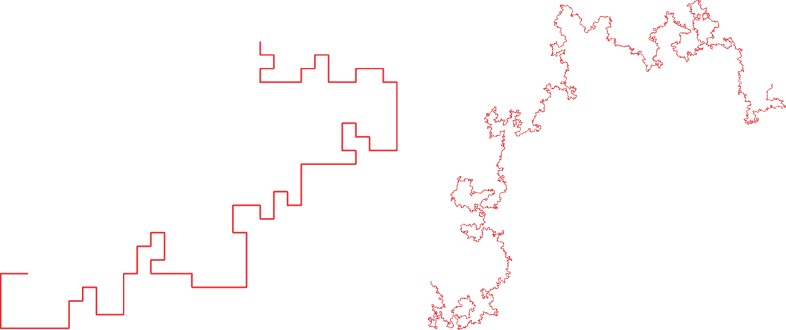
Random SAWs on the square lattice $Z^{2}$ with *n* = 10^2^ and *n* = 10^8^; image Nathan Clisby.

An *n*-step SAW on the integer lattice $Z^{d}$ is a map $\omega :\{0,1,\ldots ,n\}\to Z^{d}$, such that the Euclidean distance between *ω*(*i*) and *ω*(*i* + 1) equals 1 (nearest-neighbour steps), and such that *ω*(*i*)≠*ω*(*j*) for all *i*≠*j* (self-avoidance). Let $S_{n}$ denote the finite set of *n*-step SAWs with *ω*(0) = 0 (walk starts at origin of $Z^{d}$), and let *c*_*n*_ be its cardinality. We declare each element of $S_{n}$ to have equal probability, which must therefore be *c*^−1^_*n*_. Random *n*-step SAWs on the square lattice $Z^{2}$, with *n* = 10^2^ and 10^8^, are depicted in figure 1.

The 10^8^-step SAW in figure 1 would not be statistically distinguishable from a SAW instead on the hexagonal lattice, or on the triangular lattice, or indeed on any one of a wide variety of two-dimensional lattices. This feature is called *universality*. It is similar to the invariance principle for Brownian motion, which is the generalization of the central limit theorem that asserts that (ordinary) random walk with any finite-variance step distribution converges to Brownian motion. The search for a corresponding statement for SAW, i.e. the identification of a limiting probability law for SAW—a *scaling limit*—is one of the subject's big problems. A related and in general unproven feature is *scale invariance*: a 10^10^-step SAW, rescaled to the same size as the 10^8^-step SAW in figure 1, would be statistically indistinguishable from the 10^8^-step SAW. The scale invariance is quantified in terms of a universal *critical exponent* whose existence has not been proven in general.

#### (ii) The self-avoiding walk connective constant.

Since *c*_*n*_*c*_*m*_ counts the number of ways that an *n*-step and an *m*-step SAW can be concatenated, with the two subwalks possibly intersecting each other, we have *c*_*n*+*m*_≤*c*_*n*_*c*_*m*_. From this, it readily follows that there exists *μ* = *μ*(*d*), the *connective constant*, such that lim_*n* → ∞_*c*^1/*n*^_*n*_ = *μ* and *c*_*n*_≥*μ*^*n*^ (e.g. [1]). Good numerical estimates and rigorous bounds on the connective constant are known, but the exact value for $Z^{d}$ is not known for any *d*≥2. For SAWs defined instead on the hexagonal lattice, it has been proved that $\mu =\sqrt{2+\sqrt{2}}$ [2]. As the dimension *d* goes to infinity, there is an asymptotic expansion $\mu ∼2d-1+\sum_{n=1}^{\infty }a_{n}{\left(2d\right)}^{-n}$ with integer coefficients *a*_*n*_ whose values are known up to and including *a*_11_ [3]. The connective constant for SAWs in more general settings than $Z^{d}$ is a topic of current research [4].

Our focus here is on the asymptotic behaviour of the ratio *c*_*n*_/*μ*^*n*^, which, unlike the connective constant, is predicted to have a *universal* asymptotic behaviour.

#### (iii) Self-avoiding walk critical exponents.

There is considerable evidence from numerical studies and from arguments from theoretical physics that there exists *γ* (depending on the dimension *d*) such that

2.1 $$c_{n}∼A\mu^{n}n^{\gamma -1}\;(n\to \infty ).$$

(The symbol ∼ denotes that the ratio of left-hand and right-hand sides has limit 1.) Since *c*_*n*_≥*μ*^*n*^, necessarily *γ*≥1. The *susceptibility χ* is the generating function of *c*_*n*_:

2.2 $$\chi (z)=\sum_{n=0}^{\infty }c_{n}z^{n}\;(z\in C).$$

It has radius of convergence *z*_*c*_ = *μ*^−1^ since *c*^1/*n*^_*n*_ → *μ*. In view of (2.1), *χ* can be expected to obey

2.3 $$\chi (z)∼\frac{A^{\prime }}{{\left(z_{c}-z\right)}^{\gamma }}\;(z↑z_{c}).$$

Let *R*^2^_*n*_ be the average over $S_{n}$ of ∥*ω*(*n*)∥^2^_2_. Then *R*_*n*_ is the root-mean-square displacement of *n*-step SAWs, which is a measure of the average end-to-end distance of an *n*-step SAW. There is again considerable evidence that there exists *ν* (depending on *d*) such that

2.4 $$R_{n}∼Dn^{\nu }\;(n\to \infty ).$$

The exponent *ν* quantifies scale invariance.

This article is about *critical exponents* such as *γ*, *ν* for certain SAW and lattice spin models, with the emphasis on *γ*. There are other critical exponents that we do not discuss. The exponents are predicted to be universal, depending essentially only on the dimension of the lattice. For example, *γ* and *ν* should have the same values on the square lattice as on the hexagonal or triangular lattices, unlike the connective constant. A central problem in the subject is to prove the existence of the critical exponents and to show that they have the values listed in table 1.

**Table 1.** SAW critical exponents.

*d*	*γ*	*ν*
2	$\frac{43}{32}$	$\frac{3}{4}$
3	1.15695300(95)	0.58759700(40)
4	1 with log^1/4^	$\frac{1}{2}$ with log^1/8^
≥5	1	$\frac{1}{2}$

The rational exponents for *d* = 2 in table 1 were computed by Nienhuis [5] using non-rigorous arguments based on spin systems like the ones we discuss later. An important breakthrough came with the identification of the stochastic process SLE_8/3_ (Schramm–Loewner Evolution with parameter $\frac{8}{3}$) as the only plausible candidate for the scaling limit [6], which additionally provided an alternate explanation for the exponents $\frac{43}{32}$ and $\frac{3}{4}$. However, it remains an open problem to prove the existence of the critical exponents for *d* = 2, to prove that they have the rational values in table 1, and to prove that SLE_8/3_ truly is the scaling limit.

For *d* = 3, there is no currently known stochastic process to serve as a scaling limit for SAWs, and the best estimates for critical exponents come from numerical work. To compute the exponents, it is natural to attempt to enumerate SAWs for small *n* and then extrapolate. Fisher & Gaunt [7] found *c*_*n*_ by hand for *n*≤11, in all dimensions. More than half a century later, for *d* = 3 the enumeration has reached only *n* = 36 (table 2), which is insufficient for reliable high-precision estimation of the exponents. It is a challenging problem in enumerative combinatorics to produce a good algorithm to extend table 2 significantly. The Monte Carlo method known as the *pivot algorithm* gives more accurate estimates for critical exponents, and those appearing for *d* = 3 in table 1 are estimates using this method [8,9].

**Table 2.** *c*_*n*_ for *d* = 3, *n*≤36. The most recent values are for 31≤*n*≤36 [10].

*n*	*c*_*n*_	*n*	*c*_*n*_	*n*	*c*_*n*_
1	6	13	943 974 510	25	116 618 841 700 433 358
2	30	14	4 468 911 678	26	549 493 796 867 100 942
3	150	15	21 175 146 054	27	2 589 874 864 863 200 574
4	726	16	100 121 875 974	28	12 198 184 788 179 866 902
5	3534	17	473 730 252 102	29	57 466 913 094 951 837 030
6	16 926	18	2 237 723 684 094	30	270 569 905 525 454 674 614
7	81 390	19	10 576 033 219 614	31	1 274 191 064 726 416 905 966
8	387 966	20	49 917 327 838 734	32	5 997 359 460 809 616 886 494
9	1 853 886	21	235 710 090 502 158	33	28 233 744 272 563 685 150 118
10	8 809 878	22	1 111 781 983 442 406	34	132 853 629 626 823 234 210 582
11	41 934 150	23	5 245 988 215 191 414	35	625 248 129 452 557 974 777 990
12	198 842 742	24	24 730 180 885 580 790	36	2 941 370 856 334 701 726 560 670

The exponents *γ* = 1 and $\nu =\frac{1}{2}$ for *d*≥5 in table 1 are the same as those of simple random walk. This is summarized by the statement that the *upper critical dimension* is 4. Here is an argument to guess this: Brownian paths are two-dimensional, and since two two-dimensional objects generically do not intersect in dimensions *d* > 4, SAW should behave like a simple random walk when *d* > 4. There is a full rigorous understanding of dimensions *d*≥5. The following theorem from [11] is an example of this.

**Theorem 2.1**

*For d*≥5, *the scaling limit of SAW is Brownian motion*, *and γ* = 1 *and* $\nu =\frac{1}{2}$ *in the sense that as n* → ∞,

$$c_{n}∼A\mu^{n}\;\mathrm{and}\;R_{n}∼Dn^{1/2}.$$

Theorem 2.1 is proved using the lace expansion, which was originally introduced in [12] and has subsequently been extended to many other high-dimensional models including percolation [13,14].

For *d* = 4, the logarithms in table 1 reflect the prediction that the two asymptotic formulae in theorem 2.1 must be modified by an additional factor (log *n*)^1/4^ for *c*_*n*_ and (log *n*)^1/8^ for *R*_*n*_. We will return to such logarithmic factors later.

For *d* = 2, 3, 4, none of the entries in table 1 have been proved. In 1962, Hammersley & Welsh [15] proved the following upper bound on *c*_*n*_.

**Theorem 2.2**

*For any B* > *π*(2/3)^1/2^ *there exists an N such that for all d*≥2,

$$\mu^{n}\leq c_{n}\leq \mu^{n}\;e^{B\sqrt{n}}\;(n\geq N).$$

Shortly thereafter, for *d*≥3, Kesten improved the $\sqrt{n}$ in the exponent to *n*^2/(*d*+2)^log *n* [1,16]. For *d* = 2, the best improvements since 1962 are the replacement of *B* by *o*(1) [17], and a proof that the upper bound holds for infinitely many *n* when $B\sqrt{n}$ is replaced by *n*^0.4979^ [18]. This is slow progress in over half a century.

For *R*_*n*_, the best results are in the following theorem. The lower bound was proved in [19] and the upper bound in [20].

**Theorem 2.3**

*For d* ≥ 2,

$$\frac{1}{6}n^{2/3d}\leq R_{n}\leq o(n).$$

The lower bound fails to prove that on average the endpoint of a SAW is at least as far away as it is for simple random walk, namely *n*^1/2^, even though it appears obvious that the self-avoidance constraint must push the SAW farther than a walk without the constraint. The upper bound states that *R*_*n*_/*n* → 0 but there is no bound on the rate. In particular, it is not proved that there is a constant *C* such that *R*_*n*_≤*Cn*^0.99999^. The large gap for *d* = 2, 3, 4 between the predicted results in table 1 and those proven in theorems 2.2–2.3 is an invitation to look for more tractable models that ought to be in the same universality class as SAW. The WSAW is such a model.

### (b) Weakly self-avoiding walk

There are two versions of the WSAW: one based on discrete time (also known as the *Domb–Joyce model*) and one based on continuous time (also known as the *lattice Edwards model*). Our focus is on the latter. It differs from the SAW in two respects: (i) the underlying simple random walk model takes its steps at random times rather than after a fixed unit of time, and (ii) walks are allowed to have self-intersections but are weighted as less likely according to how much self-intersection occurs.

More precisely, let *σ*_*i*_ be a sequence of independent exponential random variables with mean 1/2*d*. Let (*X*(*t*))_*t*≥0_ denote the random walk on $Z^{d}$ which starts at the origin at time *t* = 0, waits until time *σ*_1_ and then steps immediately to a randomly chosen one of the 2*d* neighbours of the origin, then waits an amount of time *σ*_2_ until stepping to an independently randomly chosen neighbour of its current position, and so on. The *self-intersection local time* up to time *T* is the random variable

2.5 $$I(T)=\int_{0}^{T}\int_{0}^{T}{𝟙}_{X(s)=X(t)}\;ds\;dt,$$

which provides a measure of how much time the random walk has spent intersecting itself by time *T*. For fixed *g* > 0, let *c*_*T*,*g*_ = *E*(e^−*gI*(*T*)^), where *E* denotes expectation for the random walk. As a function of *T*, *c*_*T*,*g*_ is analogous to the sequence *c*_*n*_ for SAW. Every walk contributes to *c*_*T*,*g*_, but an exponential weight diminishes the role of walks with large self-intersection local time. The elementary argument which led to the existence of the connective constant generalizes to *c*_*T*,*g*_, and yields the conclusion that there exists *ν*_*c*_(*g*)≤0 such that lim_*T* → ∞_*c*^1/*T*^_*T*,*g*_ = e^*ν*_*c*_^ and *c*_*T*,*g*_≥e^*ν*_*c*_*T*^. Thus the *susceptibility*

2.6 $$\chi (g,\nu )=\int_{0}^{\infty }c_{T,g}\;e^{-\nu T}\;dT\;(\nu \in R)$$

is finite if and only if *ν* > *ν*_*c*_.

WSAW is predicted to be in the same universality class as SAW for all *g* > 0, meaning that it has the same critical exponents and scaling limits as SAW. The following theorem is an example of this, for the upper critical dimension *d* = 4 and for sufficiently small *g* > 0 [21]. It reveals that *γ* = 1 with a modification by a logarithmic correction as indicated in table 1. In the physics literature, the computation of logarithmic corrections for *d* = 4 goes back half a century [22–25]. A number of related results have been proved for the four-dimensional WSAW [26–28], all of which are consistent with the predictions for SAW.

**Theorem 2.4**

*For d* = 4 *and small g* > 0, *as t* = *ν* − *ν*_*c*_↓0,

$$\chi (g,\nu )∼A_{g}\frac{1}{t}|\log t{|}^{1/4}.$$

The proof of theorem 2.4 is based on a rigorous and non-perturbative implementation of the RG approach [29]. The RG has for decades been one of the basic tools of theoretical physics, for which Wilson was awarded the Nobel Prize in Physics in 1982. Its reach extends across critical phenomena, many-body theory, and quantum field theory. We make no attempt to refer to the vast physics literature, e.g. [30].

In a 1972 paper with the intriguing title ‘Critical exponents in 3.99 dimensions’ [31], Wilson and Fisher considered the dimension *d* as a *continuous* variable *d* = 4 − *ϵ*, and applied the RG approach to compute critical exponents in dimension 4 − *ϵ* for small *ϵ* > 0. This captures the idea that the critical behaviour can be expected to vary in a continuous manner as the dimension varies, so dimensions below *d* = 4 can be regarded as a perturbation of *d* = 4. Within physics, this has become well developed and it is found that, e.g. $\gamma =1+\frac{1}{8}\epsilon +\cdots +(\mathrm{known})\epsilon^{6}+\cdots$. Although presumably a divergent asymptotic expansion, such *ϵ*-*expansions* have been used to obtain numerical estimates of critical exponents for *d* = 3. However, from the perspective of mathematics, the dimension is not a continuous variable and this raises more questions than it answers.

### (c) Long-range walks

A different idea to move slightly below the upper critical dimension was also proposed in 1972 [32,33]. In this framework, the upper critical dimension (formerly *d* = 4) assumes a continuous value *d*_*c*_∈(0, 4). In particular, for *d* = 1, 2, 3 we can choose *d*_*c*_ = *d* + *ϵ*, and thereby study integer dimension *d* below *d*_*c*_ without the need to define the WSAW in any non-integer dimension. In our present context, this idea can be formulated in terms of walks taking long-range steps, as follows.

The long-range steps are defined in terms of a parameter *α*∈(0, 2). Let *d* = 1, 2, 3, and consider the random walk on $Z^{d}$ that takes independent steps of length *r* (in any direction) with probability proportional to *r*^−(*d*+*α*)^. This step distribution has infinite variance, a *heavy tail*. A convenient choice of such a step distribution is the fractional power −( − Δ)^*α*/2^ of the discrete Laplace operator

2.7 $${\left(\Delta f\right)}_{x}=\sum_{e\in Z^{d}:∥e{∥}_{2}=1}(f_{x+e}-f_{x}).$$

Thus we consider the random walk on $Z^{d}$ with transition probabilities

**Figure 2.**
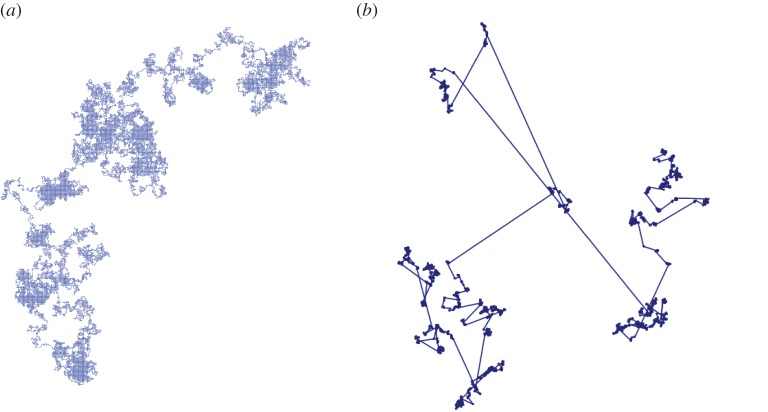
Two-dimensional 10^5^-step nearest-neighbour (*a*) and long-range (*b*, *α* = 1.1) walks (not to scale: the diameter of the left walk is about 100 times smaller than that of the right walk); image Nathan Clisby.

2.8 $$p_{x,y}=P(\text{next step to}\;y\;\;|\;\text{now at}\;x\;)\propto -{\left({\left(-\Delta \right)}^{\alpha /2}\right)}_{x,y}≍\frac{1}{∥x-y{∥}_{2}^{d+\alpha }}$$

(the notation *f* ≈ *g* means *cg* ≤ *f* ≤ *Cg* for some constants *c*, *C*). This heavy-tailed random walk converges to an *α*-stable process. The paths of an *α*-stable process have dimension *α* [34], so two such paths generically do not intersect in dimensions *d* > 2*α*. This suggests that in dimensions *d* > *d*_*c*_ = 2*α*, long-range SAW or WSAW should behave like the *α*-stable process. Figure 2 shows a two-dimensional long-range simple random walk with *α* = 1.1, next to a nearest-neighbour walk for comparison. The heavy tail of the long-range walk produces big jumps, which in turn create fewer self-intersections, thereby making it easier for a walk to be self-avoiding and lowering the upper critical dimension.

We can define a long-range model of SAW as follows. A long-range *n*-step SAW is any sequence *ω* = (*ω*(0), …, *ω*(*n*)) with $\omega (i)\in Z^{d}$ and *ω*(*i*)≠*ω*(*j*) for *i*≠*j*. Let *ω*(0) = 0. The probability of *ω* is the product $\prod_{i=1}^{n}p_{\omega (i-1),\omega (i)}$, with *p*_*x*,*y*_ given by (2.8). The following theorem [35] proves that this SAW does behave like the unconstrained long-range random walk in dimensions *d*≥1 as long as *α* < *d*/2. This is a long-range version of theorem 2.1; its proof is also based on the lace expansion. A technical point is that the theorem actually applies to a so-called *spread-out* version of the long-range SAW, a small modification.

**Theorem 2.5**

*For α*∈(0, 2) *and d* > 2*α*, *the scaling limit of spread-out long-range SAW is an α-stable process*, *and the critical exponents are γ* = 1, *ν* = 1/*α*.

However, our primary interest here is to go below *d*_*c*_ to observe scaling behaviour that is different from that of the *α*-stable process. For this, we consider WSAW and its susceptibility *χ* defined as in §2b but with the expectation *E* now with respect to the continuous-time long-range random walk. We choose $\alpha =\frac{1}{2}(d+\epsilon )$, so that *d* = *d*_*c*_ − *ϵ* is below the critical dimension *d*_*c*_ = 2*α*. The following theorem [36] gives an example of an *ϵ*-expansion. It is proved using a rigorous RG method. The restriction on *g* in the hypothesis of the theorem is used in the proof, but the statement is expected to be true for all *g* > 0. Further results are obtained in [37]. A related paper which is focused on renormalization rather than critical exponents is [38].

**Theorem 2.6**

*Let d* = 1, 2, 3. *For small ϵ* > 0, *for* $\alpha =\frac{1}{2}(d+\epsilon )$, *and for g*∈[*cϵ*, *c*′*ϵ*] *for some c* < *c*′, *there is a constant C such that as t* = *ν* − *ν*_*c*_↓0,

$$C^{-1}\frac{1}{t^{1+(1/4)(\epsilon /\alpha )-C\epsilon^{2}}}\leq \chi (g,\nu )\leq C\frac{1}{t^{1+(1/4)(\epsilon /\alpha )+C\epsilon^{2}}},\;i.e.\;\gamma =1+\frac{1}{4}\frac{\epsilon }{\alpha }+O(\epsilon^{2}).$$

## 3. Spin systems

Spin systems are basic models in statistical mechanics. We discuss two examples here: the Ising and |*φ*|^4^ models. At first sight, spin systems appear to be unrelated to SAW, but a connection will be made in §4.

### (a) Ising model

**Figure 3.**
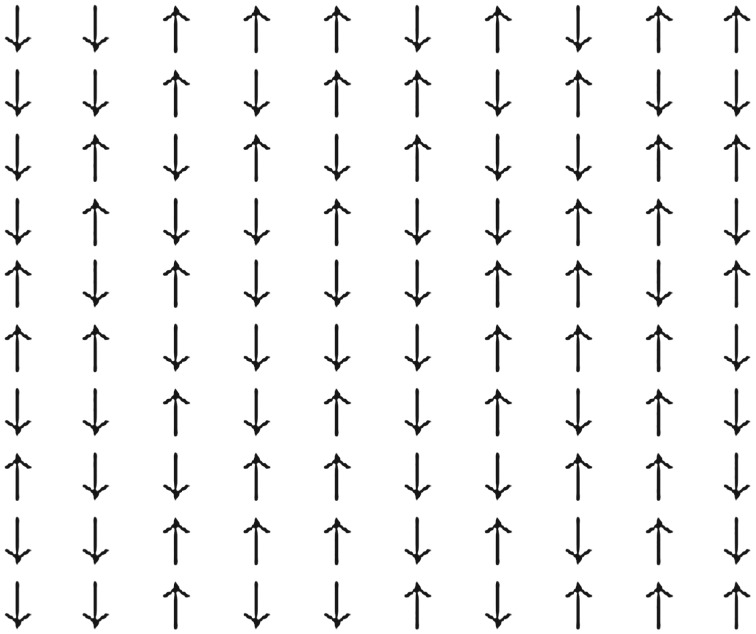
An Ising spin configuration.

The most fundamental spin system is the Ising model of ferromagnetism, which is defined as follows. Let $\Lambda \subset Z^{d}$ be a finite box. An *Ising spin configuration* on *Λ* is an assignment of +1 or −1 to each site in *Λ*, i.e. *σ* = (*σ*_*x*_)_*x*∈*Λ*_ with *σ*_*x*_∈{ − 1, 1}. An example for *d* = 2 is depicted in figure 3.

Spin configurations are random, with a probability distribution parametrized by *temperature T* and determined by the energy of *σ* which is defined to be

3.1 $$H_{\Lambda }(\sigma )=\frac{1}{2}\sum_{x\in \Lambda }\sigma_{x}{\left(-\Delta \sigma \right)}_{x}.$$

Here Δ is the discrete Laplace operator (2.7), restricted to *Λ*. Apart from an unimportant constant, *H*_*Λ*_(*σ*) is equal to $-\sum_{x∼y}\sigma_{x}\sigma_{y}$ where the sum is over all pairs of neighbouring sites in *Λ*. At temperature *T*, the probability of *σ* is given by the *Boltzmann weight*

3.2 $$P_{T,\Lambda }(\sigma )=\frac{e^{-(1/T)H_{\Lambda }(\sigma )}}{\sum_{\sigma }e^{-(1/T)H_{\Lambda }(\sigma )}}\propto e^{(1/T)\sum_{x∼y}\sigma_{x}\sigma_{y}}.$$

Thus spin configurations with more alignment between neighbouring spins are more likely than those with less alignment, and this effect is magnified for small *T* compared to large *T*.

**Figure 4.**
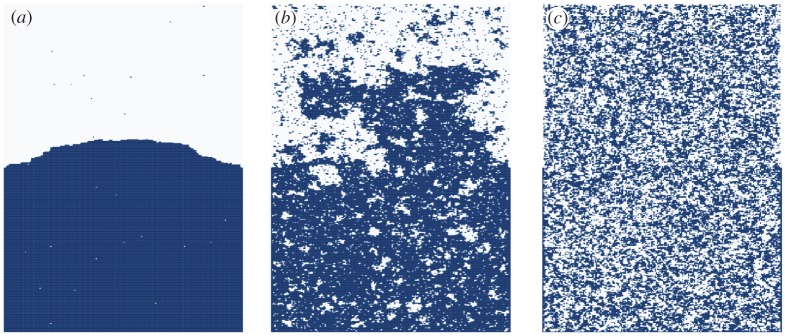
Ising configurations on a 200 × 400 box, with boundary spins fixed white on top half and dark on bottom half. (*a*) Low temperature *T* < *T*_*c*_, (*b*) critical temperature *T* = *T*_*c*_ and (*c*) high temperature *T* > *T*_*c*_.

For dimensions *d*≥2, there is a *critical* temperature *T*_*c*_ such that when *T* > *T*_*c*_ typical spin configurations are disordered, whereas for *T* < *T*_*c*_ there is long-range order. This is depicted in figure 4 where the +/ − symmetry is broken by a boundary condition. The behaviour at *T*_*c*_, and as *T* approaches *T*_*c*_, is of great current interest and there is a vast literature, particularly for *d* = 2 where the model is exactly solvable and exciting connections with SLE have been discovered, e.g. [39]. At the critical temperature, the rich geometric structure apparent in figure 4 is scale invariant. Critical exponents are rigorously known for *d* = 2 and for *d* > 4 but not for *d* = 3, although in the physics literature the conformal bootstrap has been used to compute exponents to high accuracy for *d* = 3 [40]. A recent survey of mathematical work on the Ising and related models can be found in [41].

### (b) |*φ*|^4^ model

The |*φ*|^4^ model is an extension of the Ising model in which the Ising spin *σ*_*x*_∈{ − 1, + 1} is replaced by an *n*-component vector spin $\varphi_{x}\in R^{n}$ (*n*≥1). To preserve translation invariance we replace the box used for the Ising model by one with periodic boundary conditions, i.e. *Λ* is a discrete *d*-dimensional torus. The *a priori* or *single-spin* distribution of *φ*_*x*_ is set to be proportional to e^−*V* (*φ*_*x*_)^d*φ*_*x*_, where d*φ*_*x*_ is Lebesgue measure on $R^{n}$ and

**Figure 5.**
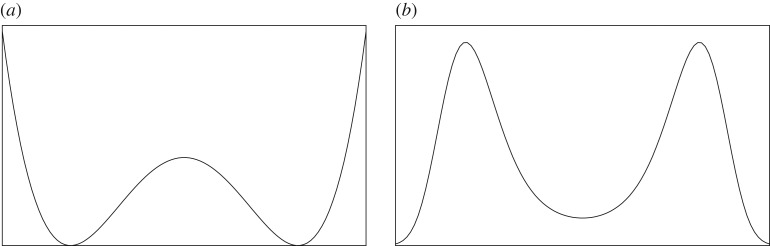
For *n* = 1, the double-well potential *V* (*a*) and single-spin density e^−*V*^ (*b*).

3.3 $$V(\varphi_{x})=\frac{1}{4}g|\varphi_{x}{|}^{4}+\frac{1}{2}\nu |\varphi_{x}{|}^{2}$$

with *g* > 0, ν∈R, and with |*φ*_*x*_| the Euclidean norm of $\varphi_{x}\in R^{n}$. We are primarily interested in *ν* < 0, in which case for *n* = 1 the potential *V* has the double-well shape of figure 5. The probability density of a spin configuration ${\left(\varphi_{x}\right)}_{x\in \Lambda }\in {\left(R^{n}\right)}^{|\Lambda |}$ is then proportional to the Boltzmann weight

3.4 $$\text{d}P_{g,\nu ,\Lambda }(\varphi )\propto e^{-\sum_{x\in \Lambda }(V(\varphi_{x})+(1/2)\varphi_{x}\cdot {\left(-\Delta \varphi \right)}_{x})}\prod_{x\in \Lambda }\text{d}\varphi_{x}.$$

For *n* = 1, spins are more likely to assume values near one of the two minima of the double well. For *n* > 1, there is a continuous set of minima. The Laplacian term in (3.4) discourages large differences between neighbouring spins and is thus a ferromagnetic interaction. For example, for *n* = 1 it encourages the spins to break the symmetry and primarily prefer one minimum over the other. Now *ν* plays the role played by the temperature *T* in the Ising model, and there is a phase transition and corresponding critical exponents associated with a critical value *ν*_*c*_(*g*) < 0. For *ν* < *ν*_*c*_, spins are typically aligned, whereas they are disordered for *ν* > *ν*_*c*_. The existence of a phase transition is proved for *d*≥3 for general *n*≥1 in [42], and for *d* = 2 and *n* = 1 in [43]; the Mermin–Wagner theorem states that there is no phase transition for *n* > 1 when *d* = 2.

The *susceptibility* is defined by

3.5 $$\chi (g,\nu )=\lim_{\Lambda ↑Z^{d}}\frac{1}{n}\sum_{x\in \Lambda }\int_{{\left(R^{n}\right)}^{|\Lambda |}}\varphi_{0}\cdot \varphi_{x}\;dP_{g,\nu ,\Lambda }(\varphi ),$$

assuming the limit exists. It represents the sum over all *x* of the correlation of the spin at 0 with the spin at *x*. If *ν* is above the critical point *ν*_*c*_ then correlations remain summable, but there is divergence at *ν* = *ν*_*c*_. The predicted behaviour of the susceptibility, as *t* = *ν* − *ν*_*c*_↓0, is

3.6 $$\chi (g,\nu )∼A_{g,n}\frac{1}{t^{\gamma }},$$

with a universal critical exponent *γ* (depending on *d*, *n*, but not *g*), and with a logarithmic correction for *d* = 4. It was proven in 1982 that *γ* = 1 for *d* > 4 [44,45].

The concept of universality was discussed in §2a. It is predicted that the one-component |*φ*|^4^ model is in the same universality class as the Ising model, and that more generally the *n*-component |*φ*|^4^ model lies in the universality class of the model in which the single spin distribution e^−*V* (*φ*_*x*_)^d*φ*_*x*_ is replaced by the uniform distribution on the sphere of radius $\sqrt{n}$ in $R^{n}$. In addition, if the nearest-neighbour interaction given by the Laplacian is replaced by any other finite-range interaction respecting the symmetries of $Z^{d}$, then the resulting model is predicted to be in the same universality class as the nearest-neighbour model.

The following theorem from [46] determines the asymptotic form of the susceptibility for *d* = 4. Its proof is via a rigorous renormalization group method. It and related work [27,28] give extensions of mathematical work from the 1980s [47–49]. (A caveat for theorems 3.1–3.2 is that the susceptibility is defined with the infinite volume limit taken through a sequence of tori of period *L*^*N*^ with fixed large *L*, as *N* → ∞.)

**Theorem 3.1**

*For d* = 4, *n*≥1, *small g* > 0, *as t* = *ν* − *ν*_*c*_↓0,

$$\chi (g,\nu )∼A_{g,n}\frac{1}{t}|\log t{|}^{\left(n+2\right)/\left(n+8\right)}.$$

To go below the upper critical dimension, we again consider a long-range version of the model, by replacing the Laplacian term *φ*_*x*_ · ( − Δ*φ*)_*x*_ in (3.4) by a term *φ*_*x*_ · (( − Δ)^*α*/2^*φ*)_*x*_ with fractional Laplacian and *α*∈(0, 2). The upper critical dimension is again *d*_*c*_ = 2*α*. Several rigorous results use the lace expansion to prove mean-field behaviour for various long-range models when *d* > *d*_*c*_, e.g. [50] for the Ising model. The following theorem from [36] concerns dimensions *d* = *d*_*c*_ − *ϵ* which lie slightly below *d*_*c*_ = 2*α*. Related results are proved in [37], and earlier mathematical papers for long-range models are [51–53].

**Theorem 3.2**

*Let d* = 1, 2, 3. *For n*≥1, *for small ϵ* > 0, *for* $\alpha =\frac{1}{2}(d+\epsilon )$, *and for g*∈[*cϵ*, *c*′*ϵ*] *for some c* < *c*′, *there is a constant C such that as t* = *ν* − *ν*_*c*_↓0,

$$\begin{array}{rl} & C^{-1}\frac{1}{t^{1+(\left(n+2\right)/\left(n+8\right))(\epsilon /\alpha )-C\epsilon^{2}}}\leq \chi (g,\nu )\leq C\frac{1}{t^{1+(\left(n+2\right)/\left(n+8\right))(\epsilon /\alpha )+C\epsilon^{2}}},\\ & \;i.e.\;\gamma =1+\frac{n+2}{n+8}\frac{\epsilon }{\alpha }+O(\epsilon^{2}). \end{array}$$

## 4. Supersymmetry and *n* = 0

Comparison of theorems 3.1–3.2 with theorems 2.4 and 2.6 reveals that when the |*φ*|^4^ theorems have the number *n* of components replaced by *n* = 0 then the WSAW statements result. This apparently curious observation is not an accident.

Indeed, de Gennes argued in 1972 [54] that the SAW model *is* the *n* = 0 version of the *n*-component spin model. Roughly speaking, his reasoning was that the susceptibility of an *n*-component spin model has a geometric representation involving a SAW and loops, with the loops weighted by *n*. When *n* is set equal to zero, only the SAW remains.

This has been a very productive observation in physics. For example, once it has been established that the |*φ*|^4^ exponent is *γ* = 1 + ((*n* + 2)/(*n* + 8))(*ϵ*/*α*) + · *s*, then the inference is made that the SAW exponent is $\gamma =1+\frac{1}{4}(\epsilon /\alpha )+\cdots$. However, from a mathematical perspective, just as it halted progress to consider non-integer dimensions *d* = 4 − *ϵ*, it is also problematic to contemplate the notion of a 0-component spin, or of a limit *n*↓0 when the dimension *n* of the spin is a natural number.

An alternate idea from physics with a similar conclusion to de Gennes's was proposed independently in 1980 by Parisi & Sourlas [55] and by McKane [56]. Their idea was that while an *n*-component boson field *φ* (usual spin) contributes a factor *n* for every loop in the geometric representation of the susceptibility, an *n*-component *fermion* field contributes −*n*. When combined, all loops cancel, leaving the SAW. From a mathematical point of view, this realization of zero components as *n* − *n* is less problematic than setting *n* = 0 or considering the limit *n*↓0, and it leads to a theorem. Some history of the mathematical work in this direction can be found in [57]. An important early step was [58], which was inspired by [59].

Fermion fields are often defined in terms of Grassmann variables, which multiply with an anti-commuting product. A fermion field can also be constructed using differential forms with their anti-commuting wedge product, and we follow this route in the following.

Given any finite set *Λ* of cardinality *M* = |*Λ*|, we consider 2*M* real coordinates and corresponding 1-forms:

4.1 $$u_{1},v_{1},\ldots ,u_{M},v_{M}\;\mathrm{and}\;\text{d}u_{1},\text{d}v_{1},\ldots ,\text{d}u_{M},\text{d}v_{M}.$$

The wedge product ∧ is associative and anti-commuting, e.g. d*u*_*x*_∧d*v*_*y*_ =  − d*v*_*y*_∧d*u*_*x*_. Let *u* = (*u*_1_, …, *u*_*M*_) and similarly for *v*. A *form* is a function of (*u*, *v*) times a product of 1-forms, or a linear combination of these. A sum of forms which each contains a product of *p* distinct 1-forms is called a *p*-form. Owing to the anti-commutativity, *p*-forms are zero if *p* > 2*M*. Also, any 2*M*-form *F* can be written uniquely as *F* = *f*(*u*, *v*)d*u*_1_∧d*v*_1_∧ · *s*∧d*u*_*M*_∧d*v*_*M*_. Integration of a 2*M*-form *F* is defined by the Lebesgue integral

4.2 $$\int F=\int_{R^{2M}}f(u,v)\text{d}u_{1}\text{d}v_{1}\cdots \text{d}u_{M}\text{d}v_{M},$$

and the integral of a *p*-form is defined to be zero if *p* < 2*M*. This definition of integration extends by linearity to arbitrary forms.

We write the 2*M* real coordinates in terms of *M* complex coordinates:

4.3 $$\phi_{x}=u_{x}+\text{i}v_{x},\;{\bar{\phi }}_{x}=u_{x}-\text{i}v_{x},\;\text{d}\phi_{x}=\text{d}u_{x}+\text{id}v_{x},\;\text{d}{\bar{\phi }}_{x}=\text{d}u_{x}-\text{id}v_{x}.$$

Let $\psi_{x}=(1/\sqrt{2\pi \text{i}})\text{d}\phi_{x}$ and ${\bar{\psi }}_{x}=(1/\sqrt{2\pi \text{i}})\text{d}{\bar{\phi }}_{x}$. The field *ϕ*_*x*_ is a two-component *boson* field on *Λ*, and *ψ*_*x*_ is a two-component *fermion* field. We define the differential forms

4.4 $$\tau_{x}=\phi_{x}{\bar{\phi }}_{x}+\psi_{x}\wedge {\bar{\psi }}_{x}\;\mathrm{and}\;\tau_{\Delta ,x}=\phi_{x}{\left(-\Delta \bar{\phi }\right)}_{x}+\psi_{x}\wedge {\left(-\Delta \bar{\psi }\right)}_{x}.$$

Smooth functions of forms are defined by Taylor expansion in $\psi ,\bar{\psi }$, which terminates as a Taylor polynomial due to the anti-commutativity. For example,

4.5 $$e^{-\sum_{x\in \Lambda }\tau_{x}}=e^{-\sum_{x\in \Lambda }\phi_{x}{\bar{\phi }}_{x}}\sum_{m=0}^{M}\frac{{\left(-1\right)}^{m}}{m!}{\left(\sum_{x\in \Lambda }\psi_{x}\wedge {\bar{\psi }}_{x}\right)}^{m}.$$

The susceptibility of the WSAW on a finite subset $\Lambda \subset Z^{d}$ is then given by the remarkable identity

4.6 $$\chi_{\Lambda }(g,\nu )=\sum_{x\in \Lambda }\int_{R^{2|\Lambda |}}e^{-\sum_{z\in \Lambda }(g\tau_{z}^{2}+\nu \tau_{z}+\tau_{\Delta ,z})}{\bar{\phi }}_{0}\phi_{x},$$

with the integral on the right-hand side evaluated according to the definition of the integral as in (4.2) after conversion of the complex coordinates to real coordinates [58]. The identity (4.6) is discussed in detail in [57], where a proof is given based on *supersymmetry*, which is a symmetry that relates the boson and fermion fields. Replacement of −Δ by ( − Δ)^*α*/2^ in the definition of *τ*_Δ,*x*_ in (4.4) and (4.6) gives a corresponding identity for the long-range model.

The right-hand side of (4.6) is reminiscent of the right-hand side of the definition of the susceptibility in (3.5). For example, the bosonic part of the exponent on the right-hand side of (4.6) matches the exponent on the right-hand side of the Boltzmann weight (3.4) for the |*φ*|^4^ model. The RG method discussed in §5 applies equally well with or without the presence of the fermion field. This allows a treatment of WSAW simultaneously with the *n*-component |*φ*|^4^ model, as the *n* = 2 − 2 = 0 case, and provides a mathematically rigorous implementation of de Gennes's idea, via the supersymmetric formulation introduced by Parisi and Sourlas and by McKane.

## 5. Renormalization group method

Theorems 2.4, 2.6 and 3.1–3.2 are proved via a rigorous RG method. Aspects of the RG method are described in this section.

### (a) Renormalization group strategy

Scaling limits in critical phenomena have the feature of scale invariance visible in the long SAW in figure 1 and in the simulation of the critical Ising model in figure 4. Wilson's brilliant strategy to exploit the scale invariance to simultaneously explain universality and provide a practical tool for the computation of universal quantities such as critical exponents can be outlined schematically as follows:

(i) Introduce a mapping, the RG map, that maps a model at one scale to a model at a larger scale. Scale invariance corresponds to a fixed point of the RG map.
(ii) A stable fixed point has a domain of attraction under iteration of the RG map. The domain of attraction is a universality class of models.
(iii) The universal properties of a scale-invariant model can be calculated from the behaviour of the RG map in the vicinity of the fixed point.

The ‘group’ operation in the term ‘renormalization group’ is the operation of composition of maps. The maps are generally not invertible, so this is a semigroup with identity rather than a group. The terminology renormalization ‘group’ has nevertheless become commonplace.

The proofs of theorems 3.1–3.2 for the |*φ*|^4^ model are based on the above strategy, with some adaptation due to lattice effects. As discussed in §4, the proofs of theorems 2.4 and 2.6 for the WSAW require relatively minor modifications of the proofs for |*φ*|^4^.

In the remainder of the paper, we flesh out the above strategy as it is employed in our context. To focus on the main ideas, we consider only the long-range *φ*^4^ model with *n* = 1 component in dimensions *d* = 1, 2, 3. The essential problem is one of a certain Gaussian integration.

### (b) Multi-scale Gaussian integration

Let *d* = 1, 2, 3. Let *Λ* be the discrete *d*-dimensional torus of period *L*^*N*^, where *L* > 1 is a fixed integer. The infinite-volume limit is achieved by *N* → ∞. Let *C* be a positive-definite |*Λ*| × |*Λ*| matrix. The Gaussian expectation $E_{C}$ with covariance *C* of a function $F:R^{|\Lambda |}\to R$ is defined by

5.1 $$E_{C}F=\frac{\int_{R^{|\Lambda |}}F(\zeta )\;e^{-(1/2)(\zeta ,C^{-1}\zeta )}\prod_{x\in \Lambda }\;d\zeta_{x}}{\int_{R^{|\Lambda |}}e^{-(1/2)(\zeta ,C^{-1}\zeta )}\prod_{x\in \Lambda }\;d\zeta_{x}}.$$

Fix *α*∈(0, 2). Let *m*^2^ > 0 and let *C* be the positive-definite |*Λ*| × |*Λ*| matrix

5.2 $$C={\left({\left(-\Delta_{\Lambda }\right)}^{\alpha /2}+m^{2}\right)}^{-1}.$$

For *g*_0_ > 0 and $\nu_{0}\in R$, let

5.3 $$Z_{0}=e^{-V_{0}(\Lambda )},\;V_{0}(\Lambda )=V_{0}(\Lambda ,\varphi )=\sum_{x\in \Lambda }\left(\frac{1}{4}g_{0}\varphi_{x}^{4}+\frac{1}{2}\nu_{0}\varphi_{x}^{2}\right).$$

The essential problem is to compute the convolution of the Gaussian expectation $E_{C}$ with *Z*_0_, namely

5.4 $$Z_{N}(\varphi )=E_{C}Z_{0}(\varphi +\zeta )=E_{C}\;e^{-V_{0}(\Lambda ,\varphi +\zeta )},$$

uniformly as *m*^2^↓0 and *N* → ∞. For example, it is an exercise in calculus to see that the finite-volume susceptibility is given by

5.5 $$\chi_{N}(g,\nu_{0}+m^{2})=\frac{1}{m^{2}}+\frac{1}{m^{4}}\frac{1}{|\Lambda |}\frac{D^{2}Z_{N}(0;𝟙,𝟙)}{Z_{N}(0)},$$

where the directions 𝟙 in the directional derivative are the constant function 𝟙_*x*_ = 1.

To evaluate (5.4), the Gaussian integration is carried out incrementally, or *progressively*, with each increment effecting integration over a single length scale. For this, we use the elementary property of Gaussian integration that if *C* = *C*′ + *C*′′ then

5.6 $$E_{C}F(\varphi +\zeta )=E_{C^{\prime \prime }}E_{C^{\prime }}F(\varphi +\zeta^{\prime \prime }+\zeta^{\prime }),$$

where on the right-hand side the inner Gaussian integral integrates with respect to *ζ*′ (holding *φ* + *ζ*′′ fixed), and the outer Gaussian integral then integrates with respect to *ζ*′′.

**Figure 6.**
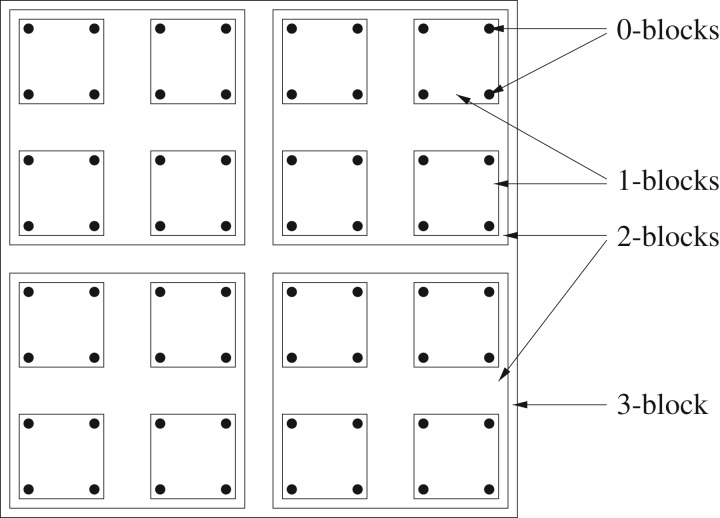
Some blocks in $B_{j}$ for *j* = 0, 1, 2, 3, with *d* = 2 and *L* = 2.

The choice of *L*^*N*^ as the period of the torus allows for the partition of the torus into disjoint *j*-*blocks* of side *L*^*j*^, for *j* = 0, 1, …, *N*. The 0-blocks are simply the points of *Λ*, and the unique *N*-block is *Λ* itself. In general, the set $B_{j}$ of *j*-blocks has *L*^(*N*−*j*)*d*^ elements. Small values of *j* are depicted in figure 6. The *scales j* = 0, 1, 2, …, *N* for the progressive integration correspond to the block side lengths *L*^0^, *L*^1^, *L*^2^, …, *L*^*N*^.

Given a covariance decomposition

5.7 $$C={\left({\left(-\Delta_{\Lambda }\right)}^{\alpha /2}+m^{2}\right)}^{-1}=\sum_{j=1}^{N}C_{j},$$

it follows from (5.4) and (5.6) that

5.8 $$Z_{N}(\varphi )=E_{C_{N}+\cdots +C_{1}}(Z_{0}(\varphi +\zeta ))=E_{C_{N}}\cdots E_{C_{2}}E_{C_{1}}(Z_{0}(\varphi +\zeta_{N}+\cdots \zeta_{1})).$$

We use a carefully constructed covariance decomposition, such that in the corresponding decomposition *ζ* = *ζ*_1_ + · *s* + *ζ*_*N*_ of the field, the *fluctuation field ζ*_*j*_ captures the fluctuations of the field *ζ* on scale *j* − 1. This is quantified by estimates on the covariances in the decomposition, which express the fact that a typical Gaussian field with covariance *C*_*j*+1_ is roughly constant on *j*-blocks and has size of order *L*^−*j*(*d*−*α*)/2^. These estimates hold until the *mass scale j*_*m*_, which is the smallest value of *j* for which *L*^*αj*^*m*^2^≥1; for scales *j* > *j*_*m*_ the covariance is smaller and the integrations for such covariances is subject to a simpler analysis. An additional finite-range property of the covariances plays an important simplifying role by making the field values in non-contiguous blocks independent [36,60–62].

In view of (5.8), we define a sequence iteratively by

5.9 $$Z_{j+1}(\varphi )=E_{C_{j+1}}Z_{j}(\varphi +\zeta )\;\mathrm{and}\;Z_{0}(\varphi )=e^{-V_{0}(\Lambda ,\varphi )}.$$

Each step in the sequence performs integration of a fluctuation field on a single scale. Then *Z*_*N*_ is the final element of the sequence, and we are interested in the limit *N* → ∞. We wish to start the sequence with *Z*_0_ defined in terms of *V*_0_ with *ν*_0_ slightly above the critical value *ν*_*c*_. However, we do not have a useful *a priori* description of *ν*_*c*_; its identification is part of the problem. To deal with this issue, we enlarge the focus, and consider a Gaussian convolution as a mapping on a space of functions of the field, defined on a suitable domain. In other words, given a covariance *C* + = *C*_*j*+1_, we write $E_{+}=E_{C_{+}}$ and define a scale-dependent map *Z*↦*Z* + by

5.10 $$Z_{+}(\varphi )=E_{+}Z(\varphi +\zeta ),$$

for integrable *Z*. Given a function *F* of the field, and given a field *φ*, we define a new function *θ*_*φ*_*F* by (*θ*_*φ*_*F*)(*ζ*) = *F*(*φ* + *ζ*). Then we can rewrite (5.10) compactly as

5.11 $$Z_{+}=E_{+}\theta Z.$$

We wish to capture the scale invariance at the critical point as a ‘fixed point’ of the mapping *Z*↦*Z*_+_. We do not achieve this literally, because of lattice effects. Indeed, the mapping is between *different* spaces, with different norms that implement rescaling. Nevertheless, the notion of a fixed point provides vital guidance.

### (c) Relevant and irrelevant monomials

The mapping *Z*↦*Z*_+_ is a transformation of one function of the field to another, and we wish to identify which are the important aspects of the map to track carefully, and which parts can be regarded as remainders.

For small *φ*, an approximation of *Z*(*φ*) involves monomials *φ*^*p*^_*x*_. The relative importance of such monomials is assessed by calculating their size when summed over a block $B\in B_{j}$, when *φ*_*x*_ is a typical Gaussian field for the covariance *C* + . For the specific choice $\alpha =\frac{1}{2}(d+\epsilon )$ in the covariance (5.2), this leads to

5.12 $$\sum_{x\in B}\varphi_{x}^{p}\approx L^{dj}({L^{-j(d-\alpha )/2})}^{p}=\left\{ \begin{array}{ll} L^{dj} & \left(p=0\right)\\ L^{\alpha j} & \left(p=2\right)\\ L^{\epsilon j} & (p=4). \end{array}\right.$$

For powers *p* > 4, a negative power of *L*^*j*^ instead occurs, so such monomials scale down as the scale is advanced. The monomials 1, *φ*^2^, *φ*^4^ are said to be *relevant* or *expanding*, while *φ*^6^, *φ*^8^, … are *irrelevant*. The relevant monomials *φ*^2^, *φ*^4^ appear already in *V*_0_. The monomial 1 plays a relatively insignificant role for the analysis of the susceptibility. Monomials containing spatial gradients need also to be considered, in general, but for the long-range model such monomials are irrelevant.

### (d) Perturbation theory

With the classification of monomials as relevant or irrelevant in mind, we treat *Z* as approximately equal to e^−*V* (*Λ*)^ with *V* given by a local polynomial $V(\Lambda )=\sum_{x\in \Lambda }(\frac{1}{4}g\varphi_{x}^{4}+\frac{1}{2}\nu \varphi_{x}^{2}+u)$ with *coupling constants g*, *ν*, *u*. We seek to find *V* + , defined with new coupling constants *g* + , *ν* + , *u* + , such that *Z* + is well approximated by e^−*V* + (*Λ*)^. Then the map *Z*↦*Z* + is approximately captured by the map *V* ↦*V* + . We refer to *V* as the *perturbative coordinate*. The term ‘perturbation theory’ refers to the evaluation of the map *V* ↦*V* + to some specific order in *V* , together with the analysis of this approximate map to compute critical exponents. We consider second-order perturbation theory here.

It is straightforward to compute $E_{+}\;e^{-\theta V(\Lambda )}$ as a formal power series in *V* to within an error of order *V*^3^. Details of a way to do this are laid out in [63]. Up to irrelevant terms, the upshot is that $E_{+}e^{-\theta V(\Lambda )}\approx e^{-V_{+}(\Lambda )}$ with $V_{+}(\Lambda )=\sum_{x\in \Lambda }(g_{+}\varphi_{x}^{4}+\nu_{+}\varphi_{x}^{2}+u_{+})$ and with the coupling constants *g* + , *ν* + , *u* + given by an explicit quadratic polynomial in *g*, *ν*, *u* with coefficients determined by the covariance *C* + .

In order to maintain the approximation of *Z* by e^−*V*^ over all scales, a critical *m*^2^-dependent choice *ν*_0_ = *ν*^*c*^_0_(*m*^2^) is required. With the wrong choice, *V* would grow exponentially and not remain small as the scale advances. As *m*^2^↓0, *ν*_0_(*m*^2^) approaches the critical value *ν*_*c*_. If we are able to control the above approximations over all scales, then we finally arrive at *Z*_*N*_(*φ*) ≈ e^−*V*_*N*_(*Λ*,*φ*)^. Substitution of this approximation into the right-hand side of (5.5) leads, after a small calculation, to

5.13 $$\chi_{N}(\nu_{0}^{c}(m^{2})+m^{2})\approx \frac{1}{m^{2}}-\frac{\nu_{N}(m^{2})}{m^{4}}.$$

The proof of theorem 3.2 in [36] verifies that the approximation (5.13) is indeed valid, and that, moreover, for small *m*^2^ > 0 the following limits hold for general *n*:

5.14 $$\lim_{N\to \infty }\nu_{N}(m^{2})=O(m^{2}\epsilon ),\;\lim_{N\to \infty }{\frac{\partial \nu_{N}(m^{2})}{\partial \nu_{0}}|}_{\nu_{0}=\nu_{0}^{c}(m^{2})}≍m^{2(\left(n+2\right)/\left(n+8\right))(\epsilon /\alpha )+O(\epsilon^{2})}.$$

Together, (5.13)–(5.14) imply the differential inequalities

5.15 $$\frac{\partial \chi }{d\nu }≍-\chi^{2-(\left(n+2\right)/\left(n+8\right))(\epsilon /\alpha )+O(\epsilon^{2})},$$

and integration then yields the statement of theorem 3.2.

Thus the proof of theorem 3.2 reduces to the validation of the approximation (5.13) with careful choice of *ν*^*c*^_0_(*m*^2^), and the computation of the limits in (5.14). The first of these two problems is significantly more difficult than the second.

A change of variables is helpful to understand the flow of coupling constants under the RG map. To incorporate the effect of the growth of relevant monomials in (5.12), it is natural to rescale the coupling constants at scale *j* as ${\hat{g}}_{j}=L^{\epsilon j}g_{j}$ and ${\hat{\nu }}_{j}=L^{\alpha j}\nu_{j}$. A further explicit change of variables $\left(\hat{g},\hat{\nu })\mapsto (s,\mu \right)$ creates a simpler triangular system. In terms of the new variables, the map *V* ↦*V* + is described by

5.16 $$s_{+}=L^{\epsilon }s(1-\beta s)$$

and

5.17 $$\mu_{+}=L^{\alpha }\left(1-\frac{n+2}{n+8}\beta s\right)\mu +\cdots ,$$

with a remainder that does not play an important role. The coefficient *β* is given in terms of the accumulated covariance $w_{k}=\sum_{i=1}^{k}C_{i}$ by

5.18 $$\beta =\beta_{j}(m^{2})=(n+8)L^{-\epsilon j}\sum_{x\in \Lambda }(w_{j+1;0,x}^{2}-w_{j;0,x}^{2}).$$

Properties of the covariance decomposition imply that, for *m*^2^ = 0, the limit *a* = lim_*j* → ∞_*β*_*j*_(0) exists; this permits *β* to be replaced by *a* in (5.16)–(5.17) up to a controlled error.

The equation *s* = *L*^*ϵ*^*s*(1 − *as*) has two fixed points: an unstable fixed point *s* = 0 and a stable fixed point $\bar{s}=(1/a)(1-L^{-\epsilon })$ which is order *ϵ*. The perturbative equations (5.16)–(5.17) can be analysed to conclude that if *s* is initially close to $\bar{s}$ then there is an initial choice of *μ*, which determines *ν*^*c*^_0_(*m*^2^), from which equations (5.16)–(5.17) can be iterated indefinitely and (5.14) holds. The requirement that *s* be chosen close to $\bar{s}$ is a requirement to be initially near the stable fixed point, and is responsible for the restriction on *g* in theorems 2.6 and 3.2.

The above analysis is based on the supposition that the approximation $E_{+}e^{-V(\Lambda ,\varphi +\zeta )}\approx e^{-V_{+}(\Lambda ,\varphi )}$ remains valid over all scales and over the entire volume *Λ*. This approximation has uncontrolled non-perturbative errors as the volume parameter *N* goes to infinity or as the field *φ* becomes large.

### (e) Non-perturbative renormalization group coordinate

The perturbative coordinate *V* is supplemented by a non-perturbative coordinate *K* which controls all errors in the above approximations. A description of *K* requires the introduction of the following concepts.

We fix a scale *j* which we drop from the notation; scale *j* + 1 is denoted by +. A *polymer* is a union (possibly empty) of blocks from B. We write P for the set of polymers, and B(X) and P(X) for the sets of blocks and polymers contained in the polymer X∈P. Let N denote the algebra of smooth functions of the field *φ*. We consider maps F:P→N, e.g. *F*(*X*) = e^−*V* (*X*)^ with $V(X,\varphi )=\sum_{x\in X}V(\varphi_{x})$. Given F,G:P→N, we define the *circle product* F∘G:P→N by

5.19 $$(F\circ G)(X)=\sum_{Y\in P(X)}F(Y)G(X\setminus Y)\;(X\in P).$$

The circle product depends on the scale, since P does. It is commutative and associative, with unit 𝟙 which takes the value 1 on the empty polymer and the value 0 on any non-empty polymer. We say that F:P→N *factorizes over blocks* if $F(X)=\prod_{B\in B(X)}F(B)$ for all X∈P, e.g. *F*(*X*) = e^−*V* (*X*)^ factorizes over blocks. If *F* and *G* both factorize over blocks then

5.20 $$(F\circ G)(X)=\prod_{B\in B(X)}(F(B)+G(B))\;(X\in P),$$

since in this case expansion of the product on the right-hand side produces the sum in (5.19).

Instead of the approximation *Z*(*Λ*) ≈ e^−*V* (*Λ*)^ used in perturbation theory, we use an exact formula

5.21 $$Z(\Lambda )=e^{-u|\Lambda |}(I\circ K)(\Lambda ).$$

Here *I* = *I*(*V* ) factorizes over blocks; it may be regarded for present purposes as *I*(*X*) = e^−*V* (*X*)^ but in fact an additional term must be included. The *K* appearing on the right-hand side of (5.21) is a non-perturbative quantity which encapsulates all errors in perturbation theory, much as the Taylor remainder formula expresses the error in a Taylor approximation. Initially, at scale 0 we have *Z*_0_(*Λ*) = e^−*V*_0_(*Λ*)^ = (e^−*V*_0_^°𝟙)(*Λ*), so (5.21) holds with *K*_0_ = 𝟙 . We seek to preserve the form of *Z* after the Gaussian expectation:

5.22 $$Z_{+}(\Lambda )=E_{+}\theta Z(\Lambda )=e^{-u_{+}|\Lambda |}(I_{+}\circ K_{+})(\Lambda ),$$

with a scale-(*j* + 1) circle product, and with the operator *θ* as in (5.11). The choice of *I* + is determined by perturbation theory. Given *any* choice of *I* + , there is a *K* + such that (5.22) holds. In fact, there are many, as the representation *Z* + (*Λ*) = e^−*u* + |*Λ*|^(*I* + °*K* + )(*Λ*) does not uniquely determine *K* + . The following proposition is a prototype for an effective choice of *K* + . For its statement, the *closure* of a polymer X∈P is defined to be the smallest polymer $\bar{X}\in P_{+}$ such that $X\subset \bar{X}$.

**Proposition 5.1**

*Suppose that I*, *I* + *factorize over blocks* $B\in B_{j}$. *For* $X\in P_{j}$, *let* $\delta I(X)=\prod_{B\in B(X)}(\theta I(B)-I_{+}(B))$. *Then*

5.23 $$E_{+}\theta (I\circ K)(\Lambda )=(I_{+}\circ {\tilde{K}}_{+})(\Lambda )$$

*with*

5.24 $${\tilde{K}}_{+}(U)=\sum_{X\in P(U)}I_{+}(U\setminus X)E_{+}((\delta I\circ \theta K)(X)){𝟙}_{\bar{X}=U}\;(U\in P_{+}).$$

Proof. By hypothesis, and by (5.20) at scale *j* with *F* = *I* + and *G* = *δI*,

5.25 $$\theta (I\circ K)=(I_{+}\circ \delta I)\circ \theta K=I_{+}\circ (\delta I\circ \theta K).$$

Let *J* = *δI*°*θK*. Since *I* + does not depend on the integration variable (which is introduced only by the operation *θ*),

5.26 $$E_{+}\theta (I\circ K)(\Lambda )=(I_{+}\circ E_{+}J)(\Lambda )=\sum_{X\in P}I_{+}(\Lambda \setminus X)E_{+}(J(X)).$$

We reorganize the sum over *X* by first summing over polymers $U\in P_{+}$ and then summing over all X∈P with closure $\bar{X}=U$. This gives

5.27 $$E_{+}\theta (I\circ K)(\Lambda )=\sum_{U\in P_{+}}I_{+}(\Lambda \setminus U)\sum_{X\in P(U)}I_{+}(U\setminus X)E_{+}(J(X)){𝟙}_{\bar{X}=U}.$$

The right-hand side is (5.23) with ${\tilde{K}}_{+}$ given by (5.24), and the proof is complete. ▪

The non-perturbative coordinate *K* must have two features: it must be *O*(*V*^3^), and it must contract as the scale advances. Each of these demands requires a norm on K:P→N; we do not describe the delicate choice of norm here [64]. The ${\tilde{K}}_{+}$ produced by Proposition 5.1 is a start, but it is insufficient as it can be shown to be *O*(*V*^2^) rather than *O*(*V*^3^), and neither is it contractive. Delicate adjustments are required to achieve these two goals [64].

On the other hand, ${\tilde{K}}_{+}$ does preserve a good factorization property. We say that polymers $X,Y\in P_{j}$ are *disconnected* if they are separated by distance at least *L*^*j*^. A polymer is *connected* if it is not the union of two disconnected polymers, and any polymer *X* partitions into connected components Comp(*X*) which are separated by at least distance *L*^*j*^. We say that F:P→N *factorizes over connected components* if $F(X)=\prod_{Y\in \mathrm{Comp}(X)}F(Y)$. The finite-range property of the covariance decomposition is the statement that *C*_*j*;*xy*_ = 0 if $∥x-y{∥}_{1}\geq \frac{1}{2}L^{j}$. This ensures that

5.28 $$E_{+}(F(X)G(Y))=E_{+}(F(X))E_{+}(G(Y))\;\mathrm{if}\;X,Y\in P_{+}\text{ are disconnected,}$$

because uncorrelated Gaussian random variables are independent.

**Figure 7.**
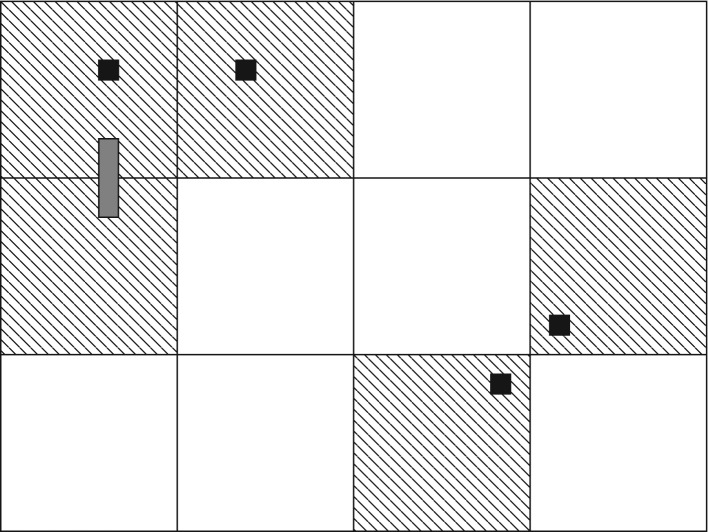
The five large shaded blocks represent *U*, which is the closure of the polymer *X* consisting of the four small dark blocks (the support of *δI*) and the small shaded rectangle (the support of *K*).

Suppose that *K* factorizes over connected components at scale *j*. It can be verified that ${\tilde{K}}_{+}$ then factorizes over connected components at scale *j* + 1, using (5.28). This factorization functions in parallel with the norm, which has the property that the norm of a product is at most the product of the norms. The geometry of the identity (5.24) defining ${\tilde{K}}_{+}(U)$ is illustrated in figure 7, which is helpful for the verification of factorization.

### (f) Renormalization group map and phase portrait

The RG map is a scale-dependent map

5.29 $$\mathrm{RG}:(s,\mu ,K)\mapsto (s_{+},\mu_{+},K_{+}),$$

defined on a suitable domain. It is defined in such a way that *K*_+_ is third order in (*s*, *μ*) if *K* is, and the *K* component is contractive under change of scale. The values of (*s*_+_, *μ*_+_) depend on *K* as well as (*s*, *μ*), and this dependence is engineered to remove the relevant parts from *K*. This extraction is responsible for the contraction of *K* under change of scale, and is indispensable for the iteration of the RG map over all scales. As long as *K* is third order, its effect on the flow of the coupling constants does not change the second-order perturbation theory that determines the asymptotic behaviour of *ν*_*N*_ and the critical exponent *γ* for the susceptibility.

**Figure 8.**
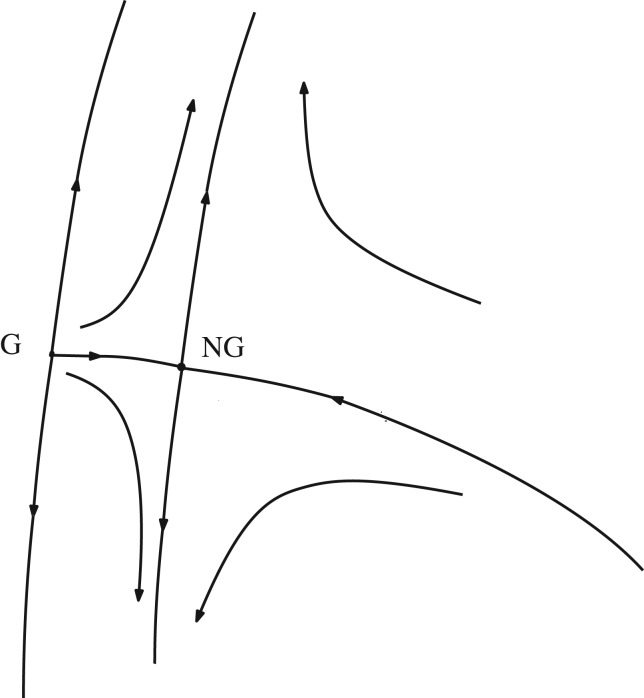
Schematic phase portrait of the dynamical system.

The RG map is used to define a map *T* on a space of sequences (*s*_*j*_, *μ*_*j*_, *K*_*j*_)_*j*≥0_, such that a fixed point of *T* corresponds to a sequence which provides a recursive solution to (5.29) for all scales *j*. The *j* = 0 value of this *global RG flow* identifies the critical point *ν*_*c*_. The RG flow is depicted schematically by the phase portrait shown in figure 8. For the long-range model with $\alpha =\frac{1}{2}(d+\epsilon )$ in dimensions *d* = 1, 2, 3, the non-Gaussian Wilson–Fisher hyperbolic fixed point is stable and the Gaussian fixed point is unstable. The critical point lies on the stable manifold from which the flow converges to the non-Gaussian fixed point. The one-dimensional unstable manifold reflects the growth of *μ* for a non-critical choice of the initial condition. For the four-dimensional nearest-neighbour model, the two fixed points merge into a single stable non-hyperbolic Gaussian fixed point.

## 6. Conclusion

The creation of a comprehensive theory of phase transitions and critical phenomena is one of the great achievements of theoretical physics during the second half of the last century. The mathematical problems posed by that theory remain a very active topic of current research. Wilson's RG approach is a cornerstone of the physical theory. Mathematical theorems based on the RG approach began to appear decades ago, but a great deal remains to be done to provide a complete and non-perturbative understanding of critical phenomena, without uncontrolled approximations. This paper concerns some recent contributions in this direction, for the WSAW and the |*φ*|^4^ lattice spin model, including a rigorous version of the *ϵ*-expansion.

## Data accessibility

This article has no additional data.

## Author profile

**Gordon Slade FRS FRSC** is Professor of Mathematics at the University of British Columbia in Vancouver, Canada, where he also obtained his PhD in 1984. Previously, he held a postdoctoral position at the University of Virginia and a faculty position at McMaster University. Slade has worked for over 30 years on problems in probability theory and statistical mechanics that arise from critical phenomena. His current research focus is on the development and application of a rigorous non-perturbative renormalization group method.
